# Supplementary material for: MALDI-TOF MS for malaria vector surveillance: A cost-comparison analysis using a decision-tree approach
Source: PLoS One. 2025 Oct 31;20(10):e0335764. doi: 10.1371/journal.pone.0335764 (PMC12578255; doi:10.1371/journal.pone.0335764)
Supplement: S1 Table — (PDF) [file pone.0335764.s001.pdf]

S1 Table: Cost analysis of reagents and consumables used in species identification by polymerase chain reaction

	Item	Size	Cost per item	Quantity used per plate	Cost per plate	Cost per sample
DNA extraction	Chelex® 100 sodium form, 200-400 Mesh	100g	401.4	0.05 g per sample	0.201 per sample	0.2012
	Ultrapure™ DNase/RNase-Free Distilled Water	500ml	18.58	100 µl per sample	0.004 per sample	0.004
	Microtube (1.5 ml)	One bag (500)	13.57	2 per sample	0.029 per tube	0.054
	Pipette tips, 200 µl	One bag (1000)	13.26	2 per sample	0.00125 per tip	0.0025
**Sub-total**						**0.26119**
PCR amplification	Ultrapure™ DNase/RNase-Free Distilled Water	500ml	18.583	500 µl	0.01875	0.0002
	Thermo-Fast 96 PCR Plate (AB-0558)	Pack of 25	133.242	1	4.75882	0.0475
	Mylar Plate Sealers (Pack of 100)	100 Sheets	118.571	1	1.18596	0.01187
	Anopheles primers	4500µl (10pmol/µl)	27.493	0.5 µl	1.8300	0.0183
	GoTaq Green Master Mix (M7123)	25 ml	791.804	500 µl	15.836	0.15871
	Pipette tips, 200 µl	One bag (1000)	13.2592	100	2.6493	0.02624
**Sub-total**						**0.26272**
Gel electrophoresis	Agarose Hi-Res Standard (100g) - 16550100	100 g	385.292	3 g	11.5584	0.11497
	Ultrapure™ 10X TBE Buffer (1L) - 15581044	1 L	22.4319	20 ml	0.44864	0.005
	100bp DNA Ladder, 500µg/ml (0.5ml) - G6951	0.5 ml	193.902	2 µl	0.77606	0.77606
	Blue/Orange Loading Dye 6X (G1881)	3ml	60.1726	3 µl	0.05999	0.05999
	Red safe staining solution (Catalog no: 21141)	1 ml	95.7263	10 µl	0.95726	0.01
	Pipette tips, 200 ul	One bag (1000)	13.2592	100	0.13247	0.00125
**Sub-total**						**0.96726**
**Grand total**						**1.49117**

Additional file 2: Cost analysis of reagents and consumables used in species identification by Sanger sequencing

**Assay**	**Item**	**Size**	**Cost per item**	**Quantity used per plate**	**Cost per plate**	**cost per sample**
	Ultrapure™ DNase/RNase-Free Distilled Water (500ML)	500ml	18.5829	500 µl	0.01875	0.00012
	Thermo-Fast 96 PCR Plate, Semi-Skirted (AB-0558)	Pack of 25	133.242	1	4.75882	0.04761
DNA amplification	Mylar Plate Sealers (Pack of 100)	100 Sheets	118.571	1	1.18596	0.01187
	Anopheles primers	4500 µl (5pmol/ µl)	27.4932	0.5 µl	0.6111	0.00612
	GoTaq Green Master Mix (M7123), 1,000 Reactions	25 ml	791.804	500 µl	15.8361	0.15834
	Pipette tips, 200 ul	1 bag (1000)	13.2592	100	2.64934	0.02649
**Sub-total**						**0.25056**
	Agarose Hi-Res Standard (100g) - 16550100	100 g	385.292	3 g	11.5584	0.1156
	Ultrapure™ 10X TBE Buffer (1L) - 15581044	1 L	22.4319	20 ml	0.44864	0.0045
Gel electrophoresis	100bp DNA Ladder, 500µg/ml (0.5ml) - G6951	0.5 ml	193.902	2 µl	0.77606	0.77556
	Blue/Orange Loading Dye 6X (G1881)	3ml	60.1726	3 µl	0.05999	0.06024
	Red safe staining solution (Catalog no: 21141)	1 ml	95.7263	10 µl	0.95726	0.00962
	Pipette tips, 200 ul	1 bag (1000)	13.2592	100	0.13247	0.00137
**Sub-total**						**0.96676**
Cleanup for PCR products	ExoSAP Express PCR Product Cleanup Reagent (75001.1.ML)	1 ml	436.579	200 µl	87.3158	1.74632
	Pipette tips, 200 ul	1 bag (1000)	13.2592	100	0.13247	0.00262
**Sub-total**						**1.74894**
	ABI PRISM Big Dye Terminator v3.1 Ready Reaction Cycle Sequencing Kit (4337456)	1,000 reactions	9,628.24	50	481.412	9.62824
	Primer	4500 ul (5pmol/ul)	27.4932	0.5 µl	0.30492	0.00612
Sequencing	Ultrapure™ DNase/RNase-Free Distilled Water (500ML)	500ml	18.5829	500 µl	0.01875	0.00012
	MicroAmp Optical 96-well reaction plate (4306737)	Pack of 20	162.622	1	8.13111	0.16258
	MicroAmp Optical adhesive covers (4311971)	pack of 100	265.197	1	2.65197	0.05299
	Pipette tips, 200 ul	1 bag (1000)	13.2592	100	0.13247	0.00262
**Sub-total**						**9.79882**
Precipitation	EDTA 0.5M, pH 8.0 (AM9261)	500ml	107.898	100 µl	0.02157	0.00037
	Sodium acetate (S7899-100ML)	100 ml	48.9878	100 µl	0.04899	0.0010

	Ethanol, Pure Absolute, >99.8% (GC) (24103-1L)	1L	26.256	1000 µl	0.02626	0.0005
	Ultrapure™ DNase/RNase-Free Distilled Water (500ML)	500ml	18.5829	3000 µl	0.11122	0.00225
	Hi-Di™ Formamide (4440753)	4 x 5ml	73.4943	1000 µl	3.67471	0.07348
	Pipette tips, 200 ul	1 bag (1000)	13.2592	100	0.13247	0.00262
**Sub-total**						**0.08036**
Capillary electrophoresis	Shipping cost to ILRI	1 box	7.28569	N/A	N/A	7.28569
	Analysis cost	Bidirectional sequencing	291.603	N/A	145.801	3.0375
**Sub-total**						**10.3232**
**Grand total**						**23.2315**
