# Supplementary material for: MALDI-TOF MS for malaria vector surveillance: A cost-comparison analysis using a decision-tree approach
Source: PLoS One. 2025 Oct 31;20(10):e0335764. doi: 10.1371/journal.pone.0335764 (PMC12578255; doi:10.1371/journal.pone.0335764)
Supplement: S3 Table — (PDF) [file pone.0335764.s003.pdf]

S3 Table: Cost analysis of reagents and consumables used in infection status determination by enzyme linked immunosorbent assay

	Item	Size	Cost per item	Quantity used per plate	Cost per plate	Cost per sample
**Sample preparation**	Micro tube (1.5 ml)	1 bag (500)	13.5458	1 per sample	0.02619 per tube	0.02619
	Casein Blocking Buffer in PBS	1L	218.279	55 ml	12.0054	0.0963
	Nonidet P40 substitute	50 ml	74.5268	125 µl	0.18585	0.0015
	Phenol Red	5 g (50 ml)	29.7234	5 µl	0.00249	0.0000
	Pipette tips, 200 ul	1 bag (1000)	13.234	1 per sample	4.36683	0.0350
**Sub-total**						**0.1545**
**Sample analysis**	PBS	1 tin (100 tablets)	19.4955	10 tablets	1.94955	0.0156
	Tween20	500 ml	71.8451	500 µl	0.07234	0.00075
	ABTS 2 component substrate (A and B)	6X450 ml	692.706	10 ml	2.57195	0.02569
	P. falciparum sporozoite ELISA reagent Kit	Donated by beiresources	0.000	0.000	0.000	0.0000
	96-well Polystyrene round plates	Pack of 50	102.891	1	2.05806	0.02058
	Pipette tips, 200 µl	1 bag (1000)	13.234	330	4.36683	0.04366
**Sub-total**						**0.11014**
**Grand total**						**0.30285**
