# Supplementary material for: MALDI-TOF MS for malaria vector surveillance: A cost-comparison analysis using a decision-tree approach
Source: PLoS One. 2025 Oct 31;20(10):e0335764. doi: 10.1371/journal.pone.0335764 (PMC12578255; doi:10.1371/journal.pone.0335764)
Supplement: S4 Table — (PDF) [file pone.0335764.s004.pdf]

S4 Table: Cost analysis of reagents and consumables used in blood meal analysis by enzyme linked immunosorbent assay (ELISA)

Assay	Item	Size	Cost per item (GBP)	Quantity used per plate (96 samples)	Cost per plate	Cost per sample
**Sample preparation**	Micro tube (1.5 ml)	1 bag (500)	13.5458	1 per sample	0.02709 per tube	0.02709
	PBS	1 tin (100 tablets)	19.4955	1 ml per sample	0.19495 per tablet	0.00195
	Pipette tips, 200 ul	1 bag (1000)	13.234	1 per sample	0.00132 per tip	0.00132
**Sub-total**						**0.03036**
**Swine**	Goat anti-Swine, IgG (H+L), HRP	0.5mg (2 ml)	138.152	2.5 µl	0.17269	0.00172
	Swine serum, 2ml	2ml	65.6709	0.5 µl	0.01641	0.00016
	PBS	1 tin (100 tablets)	19.4955	10 tablets	1.94955	0.0195
	Tween20	500 ml	71.8451	500 ul	0.07185	0.00072
	ABTS 2 component substrate (A & B)	6X450 ml	692.706	10 ml	2.57223	0.02572
	Casein Blocking Buffer in PBS	1L	218.279	55 ml	12.0054	0.12005
	96-well Polystyrene microwell plates	Pack of 50	102.891	1	2.05806	0.02058
	Pipette tips, 200 ul	1 bag (1000)	13.234	300	4.36721	0.04367
	Phenol Red	5 g (50 ml)	29.7234	5 µl	0.00297	0.00002
**Sub-total**						**0.23216**
**Goat**	Rabbit anti-Goat, IgG (H+L), HRP	0.5mg (2 ml)	69.9741	2.5 µl	0.08746	0.00087
	Goat serum, 2ml	2ml	85.2162	0.5 µl	0.0213	0.00021
	PBS	1 tin (100 tablets)	19.4955	10 tablets	1.94955	0.01950
	Tween20	500 ml	71.8451	500 µl	0.07185	0.00072
	ABTS 2 component substrate (A & B)	6X450 ml	692.706	10 ml	2.57223	0.02572
	Casein Blocking Buffer in PBS	1L	218.279	55 ml	12.0054	0.12005
	96-well Polystyrene microwell plates	Pack of 50	102.891	1	2.05806	0.02058
	Pipette tips, 200 ul	1 bag (1000)	13.234	300	4.36721	0.04367
	Phenol Red	5 g (50 ml)	29.7234	5 ul	0.00297	0.00002
**Sub-total**						**0.23136**
**Human**	Goat anti-human, IgG (H+L), HRP	0.5mg (2 ml)	205.806	2.5 µl	0.25726	0.00257
	Human serum, 2ml	2ml	473.354	0.5 µl	0.11833	0.00118
	PBS	1 tin (100 tablets)	19.4955	10 tablets	1.94955	0.01950
	Tween20	500 ml	71.8451	500 µl	0.07185	0.00072
	ABTS 2 component substrate (A & B)	6X450 ml	692.706	10 ml	2.57223	0.02572

	Casein Blocking Buffer in PBS	1L	218.279	55 ml	12.0054	0.12005
	96-well Polystyrene microwell plates	Pack of 50	102.891	1	2.05806	0.02058
	Pipette tips, 200 ul	1 bag (1000)	13.234	300	4.36721	0.04367
	Phenol Red	5 g (50 ml)	29.7234	5 µl	0.00297	0.00002
**Sub-total**						**0.23403**
	Goat anti-chicken, IgG (H+L), HRP	0.5mg (2 ml)	116.623	2.5 µl	0.14577	0.00146
	Chicken serum, 2ml	2ml	134.709	0.5 µl	0.03368	0.00034
	PBS	1 tin (100 tablets)	19.4955	10 tablets	1.94955	0.01950
	Tween20	500 ml	71.8451	500 µl	0.07185	0.00072
**Chicken**	ABTS 2 component substrate (A & B)	6X450 ml	692.706	10 ml	2.57223	0.02572
	Casein Blocking Buffer in PBS	1L	218.279	55 ml	12.0054	0.12005
	96-well Polystyrene microwell plates	Pack of 50	102.891	1	2.05806	0.02058
	Pipette tips, 200 ul	1 bag (1000)	13.234	300	4.36721	0.04367
	Phenol Red	5 g (50 ml)	29.7234	5 µl	0.00297	0.00002
**Sub-total**						**0.23206**
	Goat anti-bovine, IgG (H+L), HRP	0.5mg (2 ml)	68.602	20 µl	0.68602	0.00686
	Bovine serum, 2ml	2ml	159.406	0.5 µl	0.03985	0.00040
	DEA Buffer Solution	100ml	98.787	2 ml	1.97574	0.01976
	Phosphate Substrate Tablets 5mg/tablet	200 Tablets	171.505	2 tablets	1.71505	0.01715
**Bovine**	PBS	1 tin (100 tablets)	19.4955	10 tablets	1.94955	0.01950
	Tween20	500 ml	71.8451	500 ul	0.07185	0.00072
	Casein Blocking Buffer in PBS	1L	218.279	55 ml	12.0054	0.12005
	96-well Polystyrene microwell plates	Pack of 50	102.891	1	2.05806	0.02058
	Pipette tips, 200 ul	1 bag (1000)	13.234	300	4.36721	0.04367
	Phenol Red	5 g (50 ml)	29.7234	5 µl	0.00297	0.00002
**Sub-total**						**0.24871**
**Grand total**						**1.20869**
