# Supplementary material for: MALDI-TOF MS for malaria vector surveillance: A cost-comparison analysis using a decision-tree approach
Source: PLoS One. 2025 Oct 31;20(10):e0335764. doi: 10.1371/journal.pone.0335764 (PMC12578255; doi:10.1371/journal.pone.0335764)
Supplement: S5 Table — (PDF) [file pone.0335764.s005.pdf]

S5 Table: Cost analysis of reagents and consumables used in parity status determination by dissection

Item	Size	Cost per item	Quantity used per sample	Cost per sample
**PBS**	1 tin (100 tablets)	19.4955	**50 µL**	0.00010
**Microscope glass slides**	Pack of 50	4.27827	1 per sample	0.08557
**Glass cover slips**	pack of 200	3.79182	1 per sample	0.01896
**Pipette tips, 200 ul**	1 bag (1000 tips)	13.234	1 per sample	0.04367
**Sub-total**				**0.14829**
