# Supplementary material for: MALDI-TOF MS for malaria vector surveillance: A cost-comparison analysis using a decision-tree approach
Source: PLoS One. 2025 Oct 31;20(10):e0335764. doi: 10.1371/journal.pone.0335764 (PMC12578255; doi:10.1371/journal.pone.0335764)
Supplement: S6 Table — (PDF) [file pone.0335764.s006.pdf]

S6 Table: Cost analysis of reagents and consumables used in the MALDI-TOF MS assay

Assay	Reagent/Consumable	Size	Cost per item (USD)	Quantity used per sample/unit	Cost per sample
**Sample preparation**	Micro tube (1.5 ml)	1 bag (500)	13.5458	1 per samples	0.02744
	Glass Beads Acid Washed <106 µm	500g	402.008	0.05g per sample	0.03991
	Acetonitrile, LC/MS grade	1L	172.179	7.5 µl per sample	0.00125
	Formic Acid, LC-MS Grade	50ml	229.193	10.5 µl per sample	0.04615
	Bruker Standard Solvent	100ml	127.350	1.25 µl	0.00125
	A-Cyano-4-Hydroxycinnamic Acid (Matrix)	25g	121.151	0.0003125 g	0.00125
	Pierce water, LC-MS Grade	1L	88.8708	12 µl per sample	0.00125
	Pipette tips	1 bag (1000)	25.3703	5 tips	0.12723
**Sub-total**					**0.24572**
**Plate washing**	Trifluoroacetic Acid LC-MS Grade	50ml	187.645	80 µl per plate	0.30060
	Pierce water, LC-MS Grade	1L	88.8708	50 µl per sample	0.00499
	Pipette tips (200 µl)	1 bag (1000)	13.2340	1 tip	0.01372
	Ethanol	2.5L	78.6429	1000 ul per sample	0.03118
**Sub-total**					**0.34925**
**Grand-total**					**0.59497**
