# Supplementary material for: MALDI-TOF MS for malaria vector surveillance: A cost-comparison analysis using a decision-tree approach
Source: PLoS One. 2025 Oct 31;20(10):e0335764. doi: 10.1371/journal.pone.0335764 (PMC12578255; doi:10.1371/journal.pone.0335764)
Supplement: Attachment — Submitted filename: Article Review.docx [file pone.0335764.s008.docx]

Article Review

PLOS ONE

MALDI-TOF MS for malaria vector surveillance: a cost-comparison analysis using a decision-tree approach

The article evaluates the use of MALDI-TOF MS as an alternative to molecular methods (PCR, ELISA, sequencing) for malaria vector surveillance in Kenya. The authors employ a decision-tree analytical model, with an annual volume of 15,000 processed mosquitoes, to compare material costs, personnel costs, and turnaround time.

The results show that by adopting MALDI-TOF MS, the national surveillance program could achieve:74.48% time savings, up to 84% savings in material costs, 77% savings in labor costs, a total of 83% direct cost savings. The authors conclude that MALDI-TOF MS may bring about a paradigm shift in entomological surveillance in Africa.

The strengths of the study lie in its high practical relevance – the article addresses a central issue in malaria control: the sustainability of vector surveillance in resource-limited countries.

The described methodology is clear, employing a deterministic decision-tree model that is well justified and easy to follow.

Costs are compared in a very detailed manner – the analysis includes materials, labor, direct costs, and time, providing a comprehensive picture.

The data are applicable to public policies – the results can be used by decision-makers to optimize health budgets.

An additional important strength is the study’s global relevance – although centered on Kenya, the conclusions can be extrapolated to other malaria-endemic countries.

**Limitations and critical observations**

Incomplete MALDI-TOF database – the method still has limitations in detecting Plasmodium infections and requires confirmation by ELISA, which reduces immediate practical advantages. Moreover, the article does not specify which database was used for mosquito detection, nor whether the database is already complete or still requires validation and expansion.

Exclusion of maintenance and investment costs – the analysis ignores initial investments (equipment, staff training), which may underestimate the real costs of implementation in resource-limited countries. The MALDI-TOF device is expensive, and the amortization period compared to a classic PCR line should be specified.

Limited field validation – MALDI-TOF sensitivity and specificity for Plasmodium detection are based on controlled experiments rather than large field datasets.

Generalizability – the study relies on the Kenyan NMCP context; its exact applicability in other countries may vary depending on existing infrastructure.

In conclusion The article is well-written, rigorous, and makes a significant contribution to the field of entomological surveillance and cost optimization in malaria control. The results have practical value and are supported by a clear methodology.

**Recommendation**

Publication is recommended after minor revisions, in order to clarify development plans for the MALDI-TOF database for Plasmodium and for all mosquito species. It should also be specified that the MALDI-TOF device does not come with a built-in mosquito vector database and that such a database must be constructed separately for each region
